# Multiplexed CRISPR/Cas9-Mediated Knockout of Laccase Genes in *Salvia miltiorrhiza* Revealed Their Roles in Growth, Development, and Metabolism

**DOI:** 10.3389/fpls.2021.647768

**Published:** 2021-03-18

**Authors:** Zheng Zhou, Qing Li, Liang Xiao, Yun Wang, Jingxian Feng, Qitao Bu, Ying Xiao, Kai Hao, Meili Guo, Wansheng Chen, Lei Zhang

**Affiliations:** ^1^School of Pharmacy, Second Military Medical University, Shanghai, China; ^2^Department of Pharmacy, Changzheng Hospital, Second Military Medical University, Shanghai, China; ^3^Biomedical Innovation R&D Center, School of Medicine, Shanghai University, Shanghai, China; ^4^Research and Development Center of Chinese Medicine Resources and Biotechnology, Shanghai University of Traditional Chinese Medicine, Shanghai, China; ^5^State Key Laboratory of Subtropical Silviculture, Zhejiang A & F University, Hangzhou, China

**Keywords:** *Salvia miltiorrhiza*, laccase, CRISPR/Cas9, hairy root, development, salvianolic acid B

## Abstract

Laccases are multicopper-containing glycoproteins related to monolignol oxidation and polymerization. These properties indicate that laccases may be involved in the formation of important medicinal phenolic acid compounds in *Salvia miltiorrhiza* such as salvianolic acid B (SAB), which is used for cardiovascular disease treatment. To date, 29 laccases have been found in *S. miltiorrhiza* (*SmLACs*), and some of which (*SmLAC7* and *SmLAC20*) have been reported to influence the synthesis of phenolic acids. Because of the functional redundancy of laccase genes, their roles in *S. miltiorrhiza* are poorly understood. In this study, the CRISPR/Cas9 system was used for targeting conserved domains to knockout multiple genes of laccase family in *S. miltiorrhiza*. The expressions of target laccase genes as well as the phenolic acid biosynthesis key genes decrease dramatically in editing lines. Additionally, the growth and development of hairy roots was significantly retarded in the gene-edited lines. The cross-sections examination of laccase mutant hairy roots showed that the root development was abnormal and the xylem cells in the edited lines became larger and looser than those in the wild type. Additionally, the accumulation of RA as well as SAB was decreased, and the lignin content was nearly undetectable. It suggested that *SmLAC*s play key roles in development and lignin formation in the root of *S. miltiorrhiza* and they are necessary for phenolic acids biosynthesis.

## Introduction

**Figure 1:**
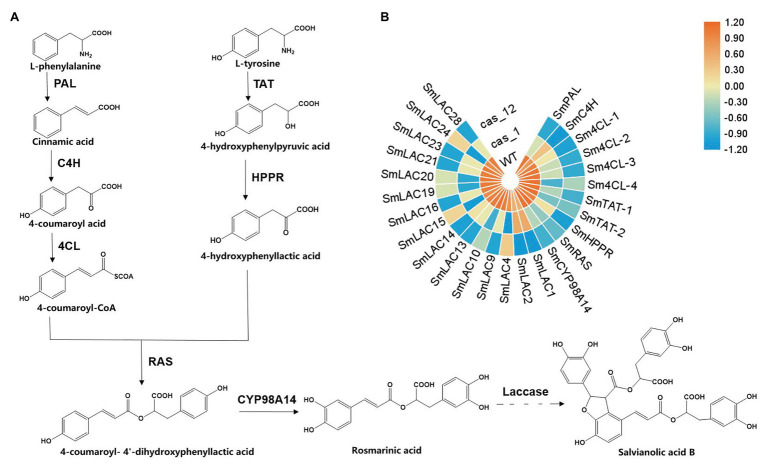
Scheme of the biosynthesis pathway of salvianolic acid B (SAB) in *Salvia miltiorrhiza* and expression profiles of key genes in transgenic hairy root lines. **(A)** Proposed phenolic acid biosynthesis pathway in *S. miltiorrhiza*. The solid line represents the verified biosynthesis process, whereas the dotted line represents the proposed biosynthetic process. **(B)** Heat maps of key genes of phenolic acid biosynthesis in the wild type (WT), *Cas1*-3, and *Cas12*-5 lines. Rows represent differentially expressed genes, and columns represent group comparisons. Blue and orange boxes represent lower and higher gene expression levels, respectively. The depth of color demonstrates the absolute value of the magnitude of the base-2 logarithmic fold change expression ratio. *PAL*, phenylalanine ammonia-lyase; *C4H*, cinnamic acid 4-hydroxylase; *4CL*, 4-coenzyme A ligase; *TAT*, tyrosine aminotransferase; *HPPR*, 4-hydroxyphenylpylpyruvate reductase; *RAS*, rosmarinic acid synthase; and *CYP98A14*, cytochrome P450 monooxygenase.

*Salvia miltiorrhiza*, also known as Danshen, is a popular and effective traditional Chinese medicine used to treat cardio-cerebral vascular diseases (Guo et al., 2014). Its pharmacological activity relies on the existence of the lipid-soluble compounds such as tanshinones, along with the water-soluble phenolic acids, including rosmarinic acid (RA), salvianic acid (Danshensu), and salvianolic acid B (SAB). Previous studies have shown that SAB, which accumulates at higher levels in roots than in other organs, may relieve ischemic cardiovascular diseases by promoting angiogenesis to improve microcirculation (Yang et al., 2016). Currently, the biosynthesis pathway of phenolic acids in *S. miltiorrhiza* has only been elucidated from phenylalanine and tyrosine to RA, and the biosynthesis of compounds with complicated structures, such as SAB, has yet to be revealed (Figure 1A; Di et al., 2013). To determine the synthetic metabolism of these phenolic acid compounds, the analysis and comparison of structural analogs to mine key enzymes is a good method.

Phenolic acids are lignin-derived products. Lignins are aromacic biopolymers derived from *p*-coumaryl, coniferyl, and sinapyl alcohol (monolignol) oxidative coupling (Tang et al., 2019). These compounds deposit secondarily thickened cell walls, in which they play the essential roles in structural mechanical support, water transport, and pathogen attack resistance (Ferrer et al., 2008; Burton et al., 2010). The polymerization of monolignols is facilitated by laccase proteins (Falade et al., 2017). Laccases are members of a multicopper oxidase family belonging to the benzenediol oxygen reductases (EC 1.10.3.2) and are also known as *p*-diphenol oxidases or urushiol oxidase (Festa et al., 2008; Wang et al., 2015). Laccases exist widely in nature and can be extracted from various species, such as bacteria, fungi, plants, and mosses (Arregui et al., 2019). Laccases are usually constructed of three Cu-oxidase domains, and they are connected as follows: Cu-oxidase_3→Cu-oxidase→Cu-oxidase_2 domain. Domains one and three house the copper sites, and the second domain often helps form a substrate-binding cleft. Highly conserved features of laccase include a copper ion bound in a Type 1 site and three more copper ions bound in atrinuclear cluster that is sometimes described as a sum of Type 2 and Type 3 sites (Solomon et al., 1996). In oxidization reactions, the substrate is first binded and oxidized at the Type 1 site in Cu-oxidase_3 domain and then transferred to a Type 2/Type 3 trinuclear copper cluster, with the release of an electron. An oxygen molecule accepts the electron, ultimately generating water (Wang et al., 2019). Because of the low substrate specificity of this gene family, most phenolic and nonphenolic molecules can be oxidized by laccases, and they are extensively applied in sewage treatment, lignin degradation, and electricity-catalyzing reactions (Viswanath et al., 2014; Chandra and Chowdhary, 2015).

In planta, laccase was first identified from the Japanese lacquer tree, *Rhus vernicifera*, and its function was revealed in a series of studies. In *Arabidopsis thaliana*, 17 putative laccase genes have been identified from the genome, and *AtLAC 4* (accession number AT2G38080), *AtLAC 11* (accession number AT5G03260), and *AtLAC 17* (accession number AT5G58910) have been verified to be related to monolignol polymerization (Berthet et al., 2011; Zhao et al., 2013). *Oryza sativa* laccase may be involved in pesticide catabolism or detoxification (Huang et al., 2016). Regarding the influence of phenolic compounds, the overexpression of *GaLAC1* (accession number AY423714.1) from *Gossypium arboretum* in *A. thaliana* resulted in an increase in sinapic acid conversion into a monolactone-type dimer (Wang et al., 2004). In *S. miltiorrhiza*, after silencing a single *SmLAC* gene, the content of RA and SAB in transgenic hairy roots was reduced (Li Q. et al., 2019).

Because of the functional redundancy of laccase genes, single suppressed gene has little influence on the plant phenotypes (Simoes et al., 2020). Therefore, it is necessary to silence or knockout multiple genes in the laccase gene family to comprehensively evaluate their roles. Due to the high homology of conserved domains in 29 laccase genes, we used the CRISPR/Cas9 system to edit conserved domains to target several laccase genes simultaneously. The editing targets were selected among 29 *SmLAC*s based on bioinformatics analysis and nucleotide and amino acid sequence alignments. Then, the targets were introduced into a binary vector to construct a single- or double-locus editing vector, which was transformed into hairy roots *via* the *Agrobacterium rhizogenes* method. The results showed that more than 20 *SmLAC*s were knocked out with this system. Quantitative real-time PCR (qRT-PCR) and RNA-Seq experiments were performed to better understand the impact of *SmLAC*s suppression, and the results demonstrated that the expression of target genes was decreased. Interestingly, the expression level of phenolic acid biosynthesis key genes was also reduced. The development of hairy roots was significantly suppressed in the gene-edited lines, and the RA and SAB contents were decreased dramatically. The examination of cross-sections of laccase mutant hairy roots showed that root development was abnormal and the xylem cells in the edited lines appeared larger and looser than those in the wild type (WT). Furthermore, lignin was nearly undetectable. In conclusion, this study showed that *SmLAC*s play important roles in development and lignin formation in the root of *S. miltiorrhiza* and they are necessary for phenolic acids biosynthesis.

## Materials and Methods

### Conserved Domain Alignment and sgRNA Design

Conserved motifs in the complete amino acid sequences of SmLACs were identified using Multiple EM software for Motif Elicitation with the setting of a maximum of 10 motifs (Bailey et al., 2006). Alignments of SmLAC gene amino acid and nucleotide sequences were performed with MEGA 5.05 software with p-distance settings. The nucleotide sequences of highly conserved domains were selected and used in the online CRISPR/Cas9 design tool CHOPCHOP^1^ to search for potential gene editing sites. Every possible editing target followed by 5'-NGG was evaluated and ranked considering the off-target possibilities and editing location. Sequences with high marks are ideal targets for editing.

### Vector Construction

Pairs of complementary oligos from selected sgRNAs were synthesized and annealed to generate dimers (Supplementary Table S1). For single-target-editing vector construction, the sgRNA1 dimer was directly cloned into the CRISPR/Cas9 system by *Bbs*I digestion. For dual-targets-editing vector construction, the sgRNA2 dimer was first cloned into the CRISPR/Cas9 system, and the cassette including AtU6 and sgRNA2 was then amplified with *Kpn*I and *Eco*RI restriction sites and subcloned into the single-target-editing vector. Finally, two CRISPR/Cas9 vectors were subcloned into the linearized pCAMBIA1300 (Cambia, Canberra, and Australia) plant expression vector by double restriction enzyme digestion and recombination.

### Plant Materials

The *S. miltiorrhiza* cultivar (RA content, 3.5–3.8% DW and SAB content, 2.8–3.0% DW) used in this study was selected and developed over several years at the Second Military Medical University. The procedures for obtaining aseptic seedlings, *Agrobacterium*-mediated transformation, and hairy root cultures were performed as previously described (Zhou et al., 2018).

### Transgenic Hairy Root Identification and Target Gene Mutation Detection

DNA was extracted from the obtained hairy roots *via* the CTAB method, and two pairs of primers were used to detect the *rolB* and *cas9* genes from the C58C1 strain, as well as binary vector by PCR amplification. The fragments surrounding *LAC10*, *LAC11*, *LAC13*, *LAC16*, *LAC21*, *LAC27*, and *LAC28* were amplified by PCR using the primers listed in Supplementary Table S1. Gene editing induced mutations was detected by aligning the sequencing chromatogram files of these PCR products with those of the WT controls. To identify the genotypes of the mutations, PCR products were cloned into a blunt zero vector (Transgen Biotech, Beijing) for sequencing.

### Relative Expression Analysis *via* Quantitative Real-Time PCR

Total RNA was extracted from samples using the EasyPure Plant RNA Kit (Transgen Biotech, China) and then reverse transcribed to produce cDNA using a TransScript First-Strand cDNA Synthesis SuperMix Kit (TransGen Biotech). qRT-PCR assays were performed as reported previously (Zhou et al., 2018). All of the primers used for qRT-PCR are listed in Supplementary Table S1.

### RNA-Seq Analysis

Total RNA was extracted from the hairy roots of *Cas1-3*, *Cas12-5*, and WT using the TRIzol reagent (TransGen Biotech, China). RNA quality was examined on a Bioanalyzer 2,100 spectrophotometer (Agilent), and the RNA was stored at −80°C. RNA samples with an RIN (RNA Intergrity Number) >8.0 were considered suitable for subsequent experiments. cDNA libraries were prepared by using the NEBNext™ Ultra Directional RNA Library Prep Kit (NEB, United States), the NEBNext Poly(A) mRNA Magnetic Isolation Module, and NEBNext Multiplex Oligos according to the manufacturer’s instructions. The products were purified and enriched by PCR to produce the final cDNA libraries and were quantified on an Agilent 2,100 spectrophotometer. The tagged cDNA libraries were pooled in equal ratios and used for 150 bp paired-end sequencing in a single lane on the Illumina HiSeqXTen platform. The fragment counts of each gene were normalized *via* the fragments per kb per million (FPKM) method.

### Histochemical Staining of Hairy Roots

Histochemical staining was performed on sections cut from the maturation zone. The hairy roots were embedded in 7% agarose before being transversely sectioned at a thickness of 50 μm using a vibratome (Leica VT1000S, Leica, Germany). The safranin O-green staining method was used to detect the deposition and composition of lignin (Bouvier et al., 2013). All sections were observed under an Olympus BX43 microscope (Japan).

### Rosmarinic Acid, Salvianolic Acid B, and Lignin Content Determination

Hairy roots of different lines were collected and dried at 40°C, and 0.1 g samples were then used to measure RA and SAB contents by high-performance liquid chromatography-tandem mass spectrometry as reported previously (Agilent 1,200, United States; Zhou et al., 2018). Lignin content was estimated spectrophotometrically as thioglycolic acid (TGA) derivative. Lignin extraction and the determination processes were similar to those described previously (Sircar and Mitra, 2009).

### Statistical Analysis

Eight independent transgenic lines from each suppression group, namely, *Cas1-2*, *Cas1-3*, *Cas1-7*, *Cas1-10*, *Cas12-1*, *Cas12-5*, *Cas12-6*, and *Cas12-9* lines were selected for subsequent analysis. The WT lines were used as controls. All parameters were expressed as the mean ± SD of three replicates. All data were derived from three biological repeats with three technical replicates. One-way ANOVA was applied and significant differences between mean values of treatments were analyzed using the Student’s *t*-test.

## Results

### Conserved Domain Alignment of SmLACs and Editing Site Selection

The complete open reading frames (ORFs) of 29 *SmLAC*s were obtained from a previous study, and each of them was analyzed for conserved domains with BLAST (Li Q. et al., 2019).

**Figure 2:**
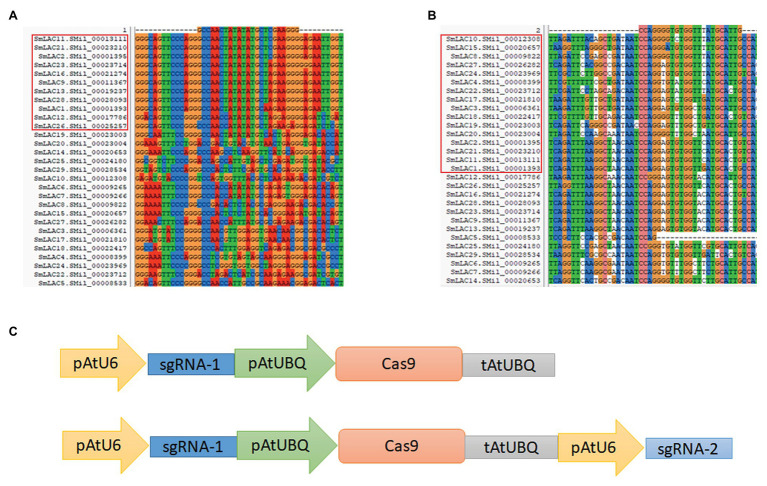
Overview of the CRISPR/Cas9 system for *SmLAC* disruption. **(A,B)** Partial sequence alignment of the first and second conserved copper domains among 29 *SmLAC*s. The target sequence and PAM sequence (GGG and TGG) are listed above. The highly conserved regions from different *SmLAC*s aligned with the editing targets are indicated (red frame). **(C)** Schematic view of two CRISPR/Cas9 knockout vectors used in this study. The cas9 protein was driven by the ubiquitin promoter, and the sgRNA was driven by the U6 promoter of *Arabidopsis*. The *Cas1* vector carried only one AtU6-sgRNA cassette, and the *Cas12* vector carried two AtU6-sgRNA cassettes to induce double mutations in *SmLAC*s.

Two conserved domain sequences were selected as target loci for editing, and the online web tool CHOPCHOP was used to search for suitable sgRNA sequences for the CRISPR/Cas9 editing system. Potential editing sites were ranked according to software scores based on several factors, such as self-complementarity and possible editing efficiency. Finally, two 20 bp gene editing sites were identified: sgRNA1: GCCAACTATATATGCTCGAA, corresponding to the conserved Cu-oxidase_3 domain, and sgRNA2: CAATGCATAAACCACACCCC, corresponding to the conserved Cu-oxidase_2 domain (Figures 2A,B).

The sgRNA1 sequence existed in 11 *SmLAC*s (*LAC1*, *LAC2*, *LAC9*, *LAC11*, *LAC12*, *LAC13*, *LAC16*, *LAC21*, *LAC23*, *LAC26*, and *LAC28*), and the sgRNA2 sequence existed in 16 *SmLAC*s (*LAC1*, *LAC2*, *LAC3*, *LAC4*, *LAC8*, *LAC10*, *LAC11*, *LAC15*, *LAC17*, *LAC18*, *LAC19*, *LAC20*, *LAC21*, *LAC22*, *LAC24*, and *LAC27*). Both the sgRNA1 and sgRNA2 sequences existed in four genes (*LAC1*, *LAC2*, *LAC11*, and *LAC21*).

### CRISPR/Cas9 Expression Vector Construction

The CRISPR/Cas9 system was provided by Jian-Kang Zhu’s laboratory at the Shanghai Center for Plant Stress Biology, Chinese Academy of Sciences. In the vector, the sgRNA was driven by the U6 promoter, and the Cas9 protein was driven by the ubiquitin promoter from *A. thaliana*. To determine the editing efficiency for multiple genes in *S. miltiorrhiza*, we constructed two vectors. To construct the single-locus knockout vector, the 20 bp sgRNA1 sequence was inserted between the *At*U6 and ubiquitin promoters. To generate the dual-site editing vector, a new cassette including the extra *At*U6 promoter and sgRNA2 was introduced into the single-locus knockout vector (Figure 2C). The whole expression cassette was subcloned into the pCAMBIA1300 plant expression vector for *A. tumefaciens*-mediated transformation. The vector carrying sgRNA1 was referred to as *Cas1*, and the vector carrying both sgRNA1 and sgRNA2 was referred to as *Cas12*. The empty pCAMBIA1300 vector was used as a control.

### Transgenic Hairy Root Identification and *SmLAC*s’ Expression Analysis

**Figure 3:**
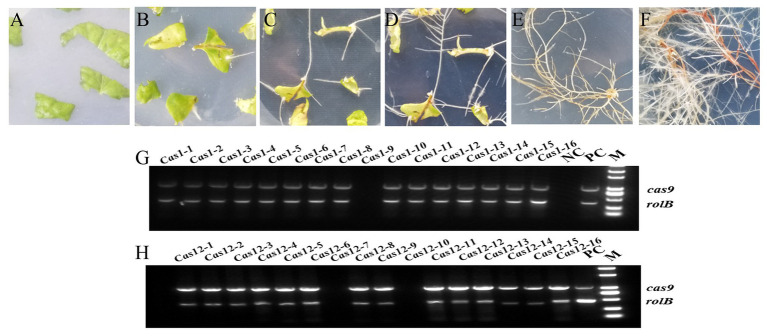
Cultivation transgenic hairy roots and identification of positive lines. **(A)** The leaf discs are spread on MS medium after *Agrobacterium*-mediated infection and undergo **(B)** callus formation, **(C)** hairy root growth, **(D)** bifurcation, **(E)** lines separation, and **(F)** growth cultivation to obtain mature roots for analysis. **(G,H)** Identification of the positive transgenic hairy roots using PCR amplification. The *rol*B gene from the *Agrobacterium rhizogenes* C58C1 strain and the *cas9* gene from the constructed vector were amplified with two pairs of primers. The empty vector line was used as a positive control (PC), and WT hairy roots were used as a negative control (NC). M indicates marker 2000.

After *Agrobacterium rhizogenes*-mediated transformation, hairy roots were regenerated from wounded young leaves and grew to maturity (Figures 3A–F). Sixteen strains of transgenic hairy roots were obtained in both the *Cas1* and *Cas12* lines, and PCR amplification was used to identify the positive transgenic hairy roots. The results showed that the CRISPR/Cas9 vector was successfully transformed into 15 transgenic hairy roots in *Cas1* lines and 14 transgenic hairy roots in *Cas12* lines (Figures 3G,H). Four transgenic hairy root lines from *Cas1* and *Cas12* lines were used in subsequent studies.

Five laccase genes from each editing domains were selected to preliminarily overview the effect of the CRISPR/Cas9 system by qRT-PCR. The results showed that the expression levels of most of the targeted genes were decreased dramatically after editing (Supplementary Figure S1). To better understand the impact of laccase editing, WT, *Cas1-3* and *Cas12-5* transgenic plants were evaluated *via* RNA-Seq experiments. A total of 57,609,184, 60,855,444, and 57,987,044 bp sequencing reads were successfully produced for WT, *Cas1-3*, and *Cas12-5*, respectively. Moreover, compared with the WT line, 3,108 and 2,666 differentially expressed genes were identified from the *Cas1-3* and *Cas12-5* lines with an FDR < 0.05 and log2|FC|>1.

The former identified 29 *SmLAC* genes as well as phenolic acid biosynthesis key genes were found on the basis of transcriptome statistics and a heat map was used to display the expression differences clearly (Figure 1B). The results showed that the expression levels of the edited laccase genes were decreased, and this result was in accordance with the qRT-PCR detection. In the *Cas1-3* lines, the expression levels of six *SmLAC*s (*LAC4*, *LAC10*, *LAC15*, *LAC19*, *LAC20*, and *LAC24*) were decreased after gene editing. Besides, nine *SmLAC*s’ expression levels of *Cas12-5* lines were also reduced (*LAC1*, *LAC2*, *LAC9*, *LAC13*, *LAC14*, *LAC16*, *LAC21*, *LAC23*, and *LAC28*). Compared with WT lines, the expression levels of genes encoding phenylalanine ammonia-lyase (*PAL*), cinnamic acid 4-hydroxylase (*C4H*): 4-coenzyme A ligase (*4CL*), tyrosine aminotransferase (*TAT*), 4-hydroxyphenylpylpyruvate reductase (*HPPR*), rosmarinic acid synthase (*RAS*), and cytochrome P450 monooxygenase (*CYP98A14*) in *SmLAC* editing lines were decreased dramatically, especially in *Cas12-5* line. These results indicate that the expression levels of most *SmLAC*s and phenolic acid biosynthesis key genes were markedly decreased in the transgenic edited lines.

To detect the mutations in the *Cas1-3* and *Cas12-5* lines, selected *SmLAC* regions (approximately 500 bp) containing the target sequences were amplified using specific primers. The purified PCR products were sequenced directly, and the corresponding sequencing chromatogram files were decoded using the degenerate sequence decoding method. *Salvia miltiorrhiza* is a diploid plant, and the CRISPR/Cas9 system can lead to two mutation types in the target genes. A simplex peak suggests that a mutation appears on both strands and can be designated a homozygous mutation. An overlapping peak indicates that a transgenic hairy root is heterozygous and contains a mutation that appears on a single strand or that different mutations occur on the two DNA strands. All the PCR products from heterozygotes were cloned into a sequencing vector to identify mutations on different strands.

Among the successfully target-edited lines, most of the mutation events consisted of one or two nucleotide deletions or insertions. However, the loss of large fragments was also detected, as found in *Cas12*-*5*-*LAC21* (Supplementary Figure S3). In most cases, the insertion or deletion of nucleotides influenced *SmLAC* translation, resulting in ORF shifts or early termination of target genes. The results indicated that it is feasible to knockout multiple targets with a single binary vector harboring several sgRNAs for gene editing in *S. miltiorrhiza*.

### Phenotypic Analysis of CRISPR/Cas9-Generated Transgenic Hairy Roots

**Figure 4:**
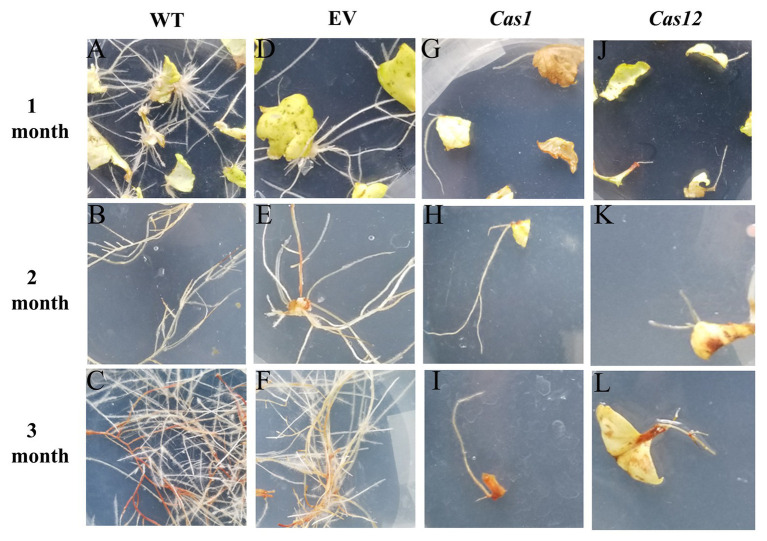
Phenotype of *SmLACs*-edited hairy roots. Hairy root growing status at 1, 2, and 3 months after *Agrobacterium*-mediated infection of **(A–C)** WT lines, **(D–F)** empty vector lines, **(G–I)** *Cas1* lines, and **(J–L)** *Cas12* lines.

To evaluate the phenotype of the transgenic hairy roots, the growth status, microstructure, and metabolite content of the WT and edited lines were collected. Compared with the thriving growth of the control lines, hairy roots in *SmLAC*s mutant lines showing retardation of root growth with the emergence of sparse root hairs (Figures 4A–F). The roots of the *Cas1* lines were thin, and there is no or only one to two lateral roots distributed along the axial root (Figures 4G–I). Moreover, the root growth of the *Cas12* lines was retarded and stopped at the elongation stage (Figures 4J–L). Additionally, the number of hairy roots extending from the callus was strikingly different among the control and gene-edited lines. Compared with the control lines, the *SmLAC*s mutant lines developed fewer regenerated roots from callus.

**Figure 5:**
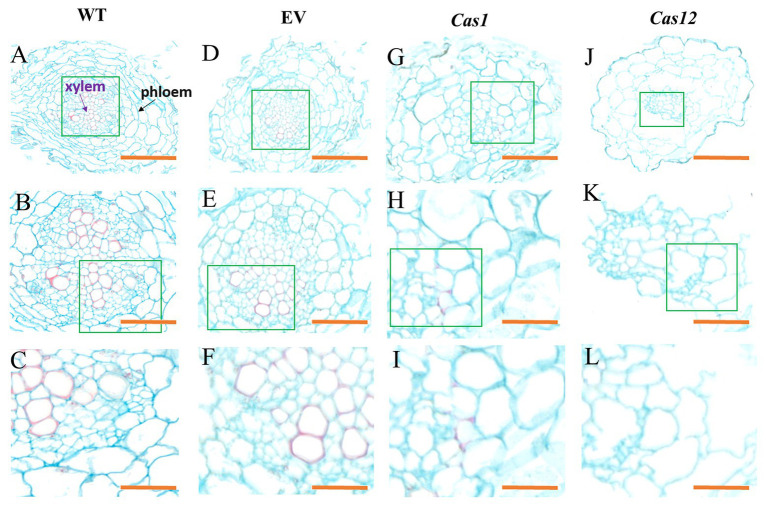
Phenotype of root microstructure in laccase mutant observed in cross-sections. The microstructure of **(A–C)** WT lines, **(D–F)** empty vector lines, **(G–I)** *Cas1* lines, and **(J–L)** *Cas12* lines. The purple arrow indicates the xylem, and the black arrow indicates the phloem. The green rectangles represent enlarged regions. Bars: (**A**,**D**,**G**,**J**) 100 μm; (**B**,**E**,**H**,**K**) 50 μm; and (**C**,**F**,**I**,**L**) 20 μm.

To investigate the root microstructure and changes in lignin after gene editing, transverse sections of the hairy roots of *Cas1-3*, *Cas12-5*, and WT plants were studied, and safranin O-green staining was performed. This staining results in the development of a red stain in the presence of lignified cell walls. It was found that lignin accumulation was rarely observable in the mutant lines compared with the WT lines. Moreover, there is no obvious lignin staining in xylem of *SmLAC*s-edited lines. Higher magnification observations of root transverse sections suggested that very few vessels were lignified in the mutants, whereas lignification was observed in most of the xylem vessels in the WT lines. Additionally, the *SmLAC*s-edited lines showed cellular structural abnormalities, reflected in differences in root cell shape and organization. The arrangement of the xylem and phloem of the *SmLAC*s-edited lines was completely different from that in the controls. In the WT and empty vector lines, the xylem vessels of the roots were radially arranged, while the phloem was arranged in concentric rings (Figures 5A–F). However, as observed in the *Cas1* lines, the xylem was arranged sparsely, and some cells in the phloem appeared to be collapsed (Figures 5G–I). According to the *Cas12* lines, disorganized vascular patterning involving xylem cells developed in regions where phloem cells were distinctly enlarged and irregular (Figures 5J–L).

**Figure 6:**
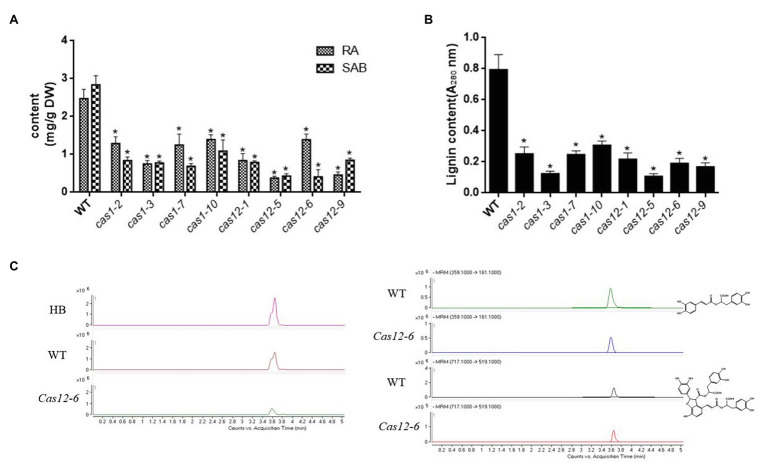
Accumulation of RA, SAB and lignin in transgenic hairy roots. **(A)** The contents of RA and SAB in transgenic hairy roots. **(B)** The contents of lignin in transgenic hairy roots. **(C)** Representative total ion chromatography (TIC) and MRM chromatography of a transgenic line (*Cas12-6*), the control line, and standard substance RA and SAB. MRM conditions for RA, SAB are as following. RA: precursor ion is 359.1, fragmentor energy is 120 V, collision energy is 14 eV, and product ion is 161.1. SAB: precursor ion is 717.1, fragmentor energy is 150 V, collision energy is 12 eV, and product ion is 519.1. All data presented here are means ± SD of three biological replicates, with error bars indicating SDs. Statistical significance was determined by the Student’s *t*-test (^*^p < 0.05).

To explore the influence of the *SmLAC*s mutations on phenolic acid synthesis, the determination of RA and SAB in regenerated hairy root extracts was performed *via* the HPLC/MS method; retention times were assessed in comparison with authentic standards as well as by searching data in the literature. The results showed that the accumulation of RA and SAB was decreased in all edited lines. The content of RA and SAB in *Cas1-3* and *Cas12-5* lines decreased considerably among the transgenic lines (Figures 6A,C). In addition, the lignin content was dramatically reduced in *SmLAC*s suppressed lines (Figure 6B). These results clearly argued that *SmLAC*s play a key role in lignin as well as phenolic acid biosynthesis.

## Discussion

Phenolic acids can be derived from the processing of lignin, and in *S. miltiorrhiza*, the biosynthesis of phenolic acids starts with L-phenylalanine and L-tyrosine, which are separately transformed into the intermediates 4-coumaroyl CoA and 3,4-dihydroxyphenyllactic acid. Rosmarinic acid synthase and cytochrome P450-dependent monooxygenase catalyze two precursors to synthesize RA. Laccase has been proposed as a key enzyme involved in the synthetic process of RA to SAB conversion (Figure 1A). The role of laccase participated in RA conversion to SAB was speculated based on the key process of lignin polymerization. This reaction involves so-called “end-wise” polymerization in which oxidized monolignol radicals undergo cross-coupling reactions with radicals formed on the free-phenolic ends of growing lignin polymers. Permutations in the regiochemistry of the radical coupling step and variations in the mode of the post-coupling quinone methide rearomatization step allow numerous possible inter-mono-meric linkages in the final lignin polymer. The SAB could be regard as the dimer of RA and it is essential to understand laccase gene family in *S. miltiorrhiza* (Supplementary Figure S2).

In previous study, although three laccase genes in *S. miltiorrhiza* (*SmLAC7*, *SmLAC20*, and *SmLAC28*) have been reported to influence phenolic acid synthesis, the roles of *SmLAC*s in physiological processes remain less clear (Li Q. et al., 2019). Here, we studied laccase function from a comprehensive perspective, focusing not only on phenolic acid metabolism but also on the alteration of physiological processes in *S. miltiorrhiza*. In this study, we reported the roles of *SmLAC*s in the alteration of lignin synthesis, which was essential for root development and phenolic acid ingredient metabolism. The retardation of hairy root growth and lateral root formation caused by CRISPR/Cas9 editing may result from the disturbance of diverse physiological and biochemical processes. The significant microstructural changes observed in transverse sections from *SmLAC*-edited lines also suggested that laccases play a key role in root development. In addition, the contents of RA and SAB as well as the expression of key genes in phenolic acid biosynthesis pathway were decreased dramatically, suggesting that *SmLAC* genes are involved in phenolic acid biosynthesis.

The laccase genes in *S. miltiorrhiza* can be clustered into seven groups according to previous study (Li Q. et al., 2019). In this research, the target of sgRNA1 belonged to group VII and the sgRNA2 belonged to I, II, III, IV, and V groups. The results showed that the accumulation of lignin and phenolic acids in most of *cas12* lines was lower than that in *cas1* lines. Additionally, the ratio of SAB to RA seemed to be lower than that of WT lines if laccase was involved in dimerization of RA to SAB. However, the contents of RA and SAB among the transgenic lines were not as expected. Furthermore, the growing status of *cas12* lines seemed to be poorer than *cas1* lines. These phenotypes indicated that the functions of *SmLACs* are redundant and their roles in different *SmLAC*s groups are cross-functional. It is meaningful to figure out their function in following studies.

Despite the high degree of redundancy of laccase genes in *S. miltiorrhiza*, it is difficult to identify lignin-specific genes (Li C. et al., 2019). It is time consuming to generate multiple mutants to study the function of multiple genes. In gene family function studies in particular, the function of a single gene might be redundant, and multiple genes should be knocked out to explore their phenotypes and roles. Therefore, this study also provides new gene editing methods to study laccase gene family in *S. miltiorrhiza*. In previous studies, the CRISPR/Cas9 system as reported to target a single gene locus at a time, and this study performed dual-locus editing in *S. miltiorrhiza* (Li et al., 2017; Zhou et al., 2018; Deng et al., 2020). This work will facilitate the application to a new strategy for functional studies, targeting multiple genes and gene families in *S. miltiorrhiza*.

**Figure 7:**
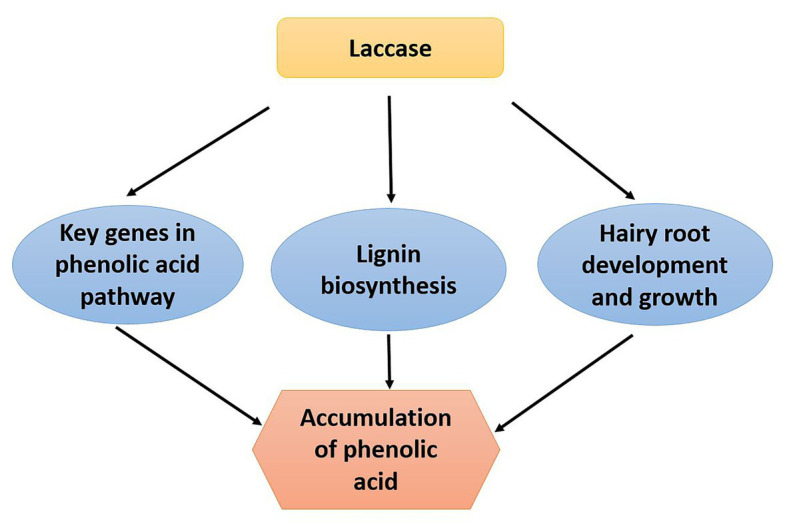
The mechanistic model depicting *S. miltiorrhiza* laccase gene family regulates phenolic acid accumulation. The laccase gene family of *S. miltiorrhiza* may regulate accumulation of phenolic acid by influencing expression level of phenolic acid biosynthesis key genes, lignin biosynthesis, and hairy root growth and development.

In conclusion, we revealed the function of laccase in *S. miltiorrhiza*. The laccase family may influence expression level of phenolic acid biosynthesis key genes, lignin biosynthesis, and hairy root growth and development resulting in the accumulation of phenolic acid content (Figure 7). The results provide a solid ground for further exploring laccases and uncover multiply functions of laccase in *S. miltiorrhiza*.

## Data Availability Statement

The datasets presented in this study can be found in online repositories. The names of the repository/repositories and accession number(s) can be found in the article/Supplementary Material.

## Author Contributions

ZZ, MG, WC, and LZ conceived and designed the entire research plans. ZZ, QL, JF, and LX performed most of the experiments. QL, YW, YX, QB, and KH provided technical assistance to ZZ. ZZ and LZ wrote the manuscript. MG and WC helped with the organization and editing. All authors contributed to the article and approved the submitted version.

### Conflict of Interest

The authors declare that the research was conducted in the absence of any commercial or financial relationships that could be construed as a potential conflict of interest.

## Footnotes

^1^http://chopchop.cbu.uib.no/
