# Supplementary material for: Multiplexed CRISPR/Cas9-Mediated Knockout of Laccase Genes in Salvia miltiorrhiza Revealed Their Roles in Growth, Development, and Metabolism
Source: Front Plant Sci. 2021 Mar 18;12:647768. doi: 10.3389/fpls.2021.647768 (PMC8014014; doi:10.3389/fpls.2021.647768)
Supplement: Supplementary file 1 [file Data_Sheet_1.pdf]

Figure S1 Expression profiles of *SmLAC*s in transgenic hairy root lines.

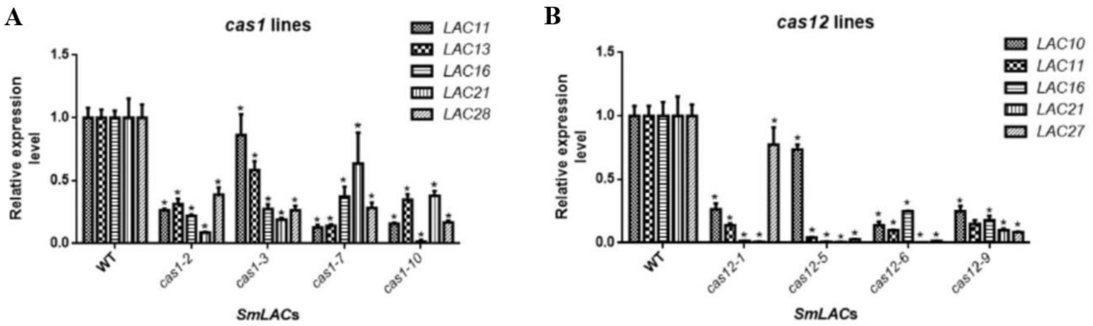

(A) Expression levels of five laccase genes (*SmLAC11*, *SmLAC13*, *SmLAC16*, *SmLAC21*, *SmLAC28*) in *Cas1* lines. (B) Expression levels of five laccase genes (*SmLAC10*, *SmLAC11*, *SmLAC16*, *SmLAC21*, *SmLAC27*) in *Cas12* lines. All data are means of three replicates, with error bars indicating standard deviations. *SmActin* was used as internal control. The values are means ± S.D. of triplicate analysis, *P < 0.05.

Figure S2 Catalytic mechanism of laccase in dimer biosynthesis.

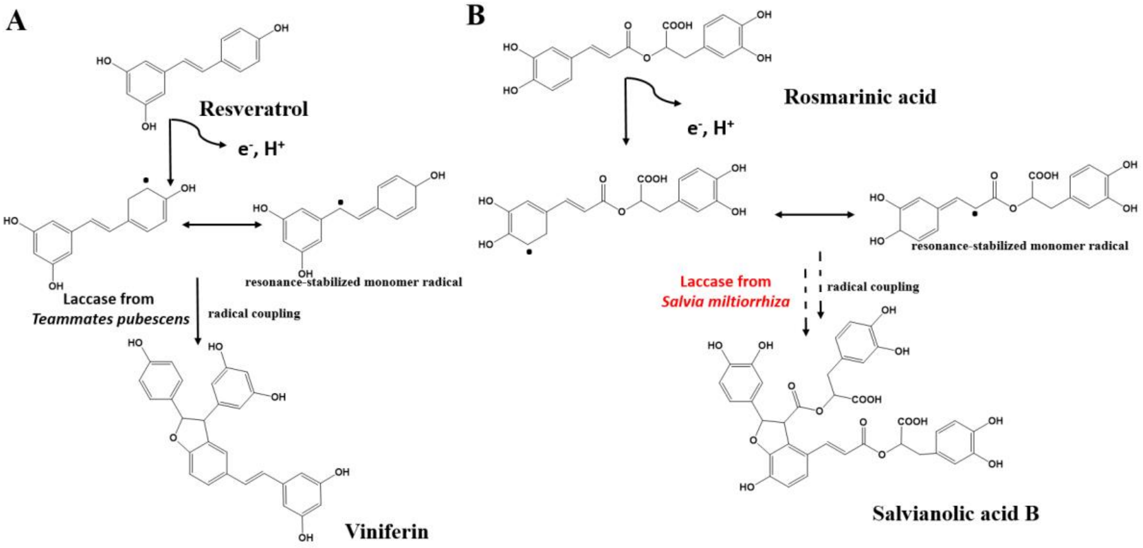

(A) The resveratrol can be catalyzed to viniferin by *Teammates pubescens* laccase. (B) Proposed salvianolic acid B biosynthesis mechanism from rosmarinic acid catalyzed by *Salvia miltorrhiza* laccase.

Figure S3 Schematic of the sgRNA design and editing results.

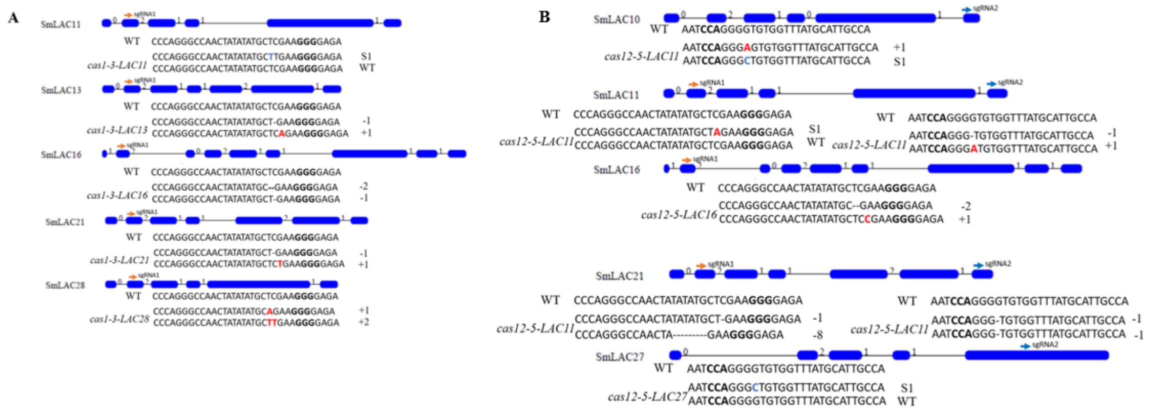

(A) Sequence analysis of the two alleles of the *Cas1-3* hairy root line. (B) Sequence analysis of the two alleles of the *Cas12-5* hairy root line. Black bold text, PAM sequence. Red bold text, nucleotides insertion. Blue bold text, nucleotides substitution. The change in the number of nucleotides is shown on the right of each allele sequence. The '+' suggests an insertion and the '–' indicates a deletion. The 'S' represents the substitution of nucleotide. The 'WT' represents wild type line sequence. 0, 1 and 2 indicate intron phases.

Table S1 Primers used in this study

Primers	Sequence (5'-3')	Purpose assay
sgRNA1-F	GATTGCCAACTATATATGCTCGAA	CRISPR/cas9 vector construction
sgRNA1-R	AAACGGTTGATATATACGAGCTTC	
sgRNA2-F	GATTGCAATGCATAAACCACACCCC	
sgRNA2-R	AAACGTTACGTATTTGGTGTGGGGC	
qLAC10-F	GGTGGAATGATGATATAC	quantitative real-time PCR
qLAC10-R	ACTCAACTTCAATGTATC	
qLAC11-F	GCATTGAAGTTGAGTGTG	
qLAC11-R	AAGTTGTGGTCCTTGATT	
qLAC13-F	GCAAGGTCATAAGTCATT	
qLAC13-R	TTCCGAGTTATAGTCCAA	
qLAC16-F	ATGGAGCACTATCAGATT	
qLAC16-R	GTCTCTAACGATGAACAC	
qLAC21-F	AGCCATCGTCATTCTACC	
qLAC21-R	ATCATCATTCCACCACTCT	
qLAC27-F	TGGATTCTTGGATGATTAGG	
qLAC27-R	AACTTGTAATGGCGAACT	
qLAC28-F	ACGCTATAATCTCATTGAC	
qLAC28-R	AATCTGATAGTGCTCCAT	
LAC10-F	ATGGCAGTCACAACCACTGC	Laccase gene amplification
LAC10-R	GCACCGAGGGTAATCAGCTGG	
LAC11-F	ATGTTTTTTCATAAGGTTTTCA	
LAC11-R	ATTGTTGAGGCTGTATGGGC	
LAC13-F	ATGGTTTCTTATAGGAAGCT	
LAC13-R	AATCCTAAATAATAATTGTTGAGG	
LAC16-F	ATGCAGGAACAAGATTTTCGA	
LAC16-R	AATCCTAAACAATAATTGTTG	
LAC21-F	ATGCACACCACTGAAAAAATGTTT	
LAC21-R	AATTGTTGAGGCTGCAGGGGG	
LAC27-F	ATGGATTCTTGGATGATTAGGG	
LAC27-R	TGAGCATGTGGGTAAATCTTGAG	
LAC28-F	ATGTTTTCTTATAGGAAGCTT	
LAC28-R	AATCCTAAACAATAATTGTTG	
rolB-F	GCTCTTGCAGTGCTAGATTT	Positive transgenic lines identification
rolB-R	GAAGGTGCAAGCTACCTCTC	
cas9-F	ATGGACTATAAGGACCACGA	
cas9-R	TTGGTCATCCGCTCGATGAAGCT	

Table S2 Identified phenolic acid biosynthesis key genes' information in transcriptome statistics.

Gene Name	Accession Number	Gene ID in Transcriptome Statistics
SmPAL phenylalanine ammonia-lyase [*Salvia miltiorrhiza*]	ABR14606.1	TRINITY_DN54449_c0_g1_i1
SmC4H cinnamate 4-hydroxylase [*Salvia miltiorrhiza*]	ABC75596.1	TRINITY_DN53631_c0_g1_i4
Sm4CL-1 4-coumarate:coenzyme A ligase 1 [*Salvia miltiorrhiza*]	AAP68990.1	TRINITY_DN55567_c0_g4_i1
Sm4CL-2 4-coumarate:coenzyme A ligase 4 [*Salvia miltiorrhiza*]	AGW27201.1	TRINITY_DN28291_c0_g1_i1
Sm4CL-3 4-coumarate:coenzyme A ligase 7 [*Salvia miltiorrhiza*]	AGW27197.1	TRINITY_DN52540_c1_g3_i1
Sm4CL-4 4-coumarate:coenzyme A ligase 9 [*Salvia miltiorrhiza*]	AGW27199.1	TRINITY_DN49021_c0_g1_i1
SmTAT-1 Tyrosine aminotransferase [*Salvia miltiorrhiza*]	ABC60050.1	TRINITY_DN50798_c0_g1_i8
SmTAT-2 tyrosine aminotransferase 2 [*Salvia miltiorrhiza*]	AGW27212.1	TRINITY_DN58698_c0_g1_i1
SmHPPR putative hydroxyphenylpyruvate reductase [*Salvia miltiorrhiza*]	AAZ67354.1	TRINITY_DN51154_c0_g1_i1
SmRAS rosmarinic acid synthase [*Salvia miltiorrhiza*]	ADA60182.1	TRINITY_DN55819_c0_g1_i3
SmCYP98A14 putative cytochrome P450 [*Salvia miltiorrhiza*]	ADP00279.1	TRINITY_DN52935_c0_g1_i2
